# The impact of sitting time and physical activity on mental health during COVID-19 lockdown

**DOI:** 10.1007/s11332-021-00791-2

**Published:** 2021-06-10

**Authors:** Matthew Pears, Susanna Kola-Palmer, Liane Beretta De Azevedo

**Affiliations:** 1 Department of Psychology, University of Huddersfield, Huddersfield, HD1 3DH West Yorkshire UK; 2 Department of Allied Health Professionals, Sports, and Exercise, University of Huddersfield, Queensgate, Huddersfield, HD1 3DH West Yorkshire UK

**Keywords:** Pandemic, Depression, Anxiety, Wellbeing, Sedentary behaviour, Regression

## Abstract

**Objective:**

The primary aim of this study was to investigate the association between physical activity (PA) and sitting time on adults’ mental health (i.e., depression, anxiety and wellbeing) and the influence of mediators and confounders.

**Methods:**

An online survey was disseminated in the UK between May and June 2020. A total of 284 participants (33.5 ± 12.4 years) self-reported their PA, sitting time and mental health through validated questionnaires.

**Results:**

Multiple stepwise regression analysis revealed that being of younger age, female, on a lower income, with one or more comorbid health conditions, with a previous diagnosis of mood disorder and increased sitting time independently correlated with higher depression scores (*F* (13,219) = 12.31, *p* < 0.001), and explained 42% of the variance. Similar results were found for wellbeing where socio-demographic, health outcomes and sitting time influenced the subjective wellbeing (*F* (14,218) = 5.77, *p* < 0.001, 27% variance), although only socio-demographic and health outcomes contributed to the variation in anxiety score (*F* (13,219) = 7.84, *p* < 0.001, 32% variance). PA did not explain variation when sitting time was taken into account in any of the models. Combined analysis revealed that participants with lower sedentary time (< 8 h) and with both low or moderate and high PA presented a significantly lower depression score [low PA: (*B* = −2.7, 95% CI −4.88, −0.52); moderate and high PA (*B* = −2.7, −4.88, −0.52)].

**Conclusion:**

Sitting time was strongly associated with adverse mental health during COVID-19 lockdown and should be considered in future public health recommendations.

## Introduction

Since the end of 2019, a life-threatening strain of the severe acute respiratory syndrome coronavirus (COVID-19) has caused extreme global public health concern. The person-to-person transmission of the infection led global governments to deploy policies of isolation and social distancing to control the pandemic [1, 2]. This caused a spike in cases of stress disorders [3], depression, anxiety [4, 5] , and behavioural changes towards harmful health consequences in countries where COVID-19 lockdown was in place [6, 7]. Currently, depression is the leading cause of disability around the world, affecting approximately 264 million people [8]. Anxiety is also one of the most prevalent psychiatric disorder with reports that one-third of the population may suffer from anxiety during their lifetime [9]. Social isolation, distancing, and loneliness have significant implications for psychological symptoms including depression, anxiety, and reduction in their quality of life [10–12]. This can increase cardiovascular risk and all-cause mortality [13, 14]. Furthermore, increased anxiety and depressive cognitions can dysregulate the immune and endocrine systems and compromises body immunity [15, 16]. These effects hinder the ability to combat COVID-19, as the disease can further suppress the immune system responses [17]. Indeed, a recent study highlighted that people with suspected COVID-19 symptoms had higher depression likelihood and lower health-related quality of life [18].

There has been strong evidence that physical activity is an effective strategy in reducing anxiety, depression, and negative mood [19–21], which has been confirmed by systematic reviews of prospective studies [22] and randomised controlled trials [23]. The multiple benefits of physical activity for physical and mental wellbeing was recognised by the UK government, when the first guidance of the lockdown was published [24], which included exercising outdoors as one of the few reasons why people could leave home [25]. However, evidence of physical activity behaviour and the effect on mental health during lockdown has only started to be explored by the literature. The recent literature consistently showed a decrease in physical activity and an increase in sitting time [26] with an effect on wellbeing [27]. A survey from Australia reported that changes in physical activity were associated with higher depression, anxiety, and stress symptoms during the lockdown period [22, 28]. Meanwhile, studies from America reported that participants who reduced physical activity and increased screen time from pre and post COVID-19 increased the chances of depression loneliness and stress [29], while another reported a positive effect of light activity on mental health outcomes [30]. Finally, a recent UK online survey reported that mental health outcomes (depression, anxiety, and mental wellbeing) were negatively associated with moderate to vigorous physical activity per day [31].

Until now, most of the studies have focused on physical activity instead of sedentary behaviour, and there is evidence that imposing sedentary behaviour has a negative impact on mental wellbeing even in a short time period of 7 days [32]. However, it has not yet known if physical activity could mediate the adverse effects of sedentary behaviour on mental health during lockdown restriction. Similarly, there is a need for further understanding of the impact of cofounders, such as socioeconomic status and other pre-existing health conditions which were already seen as predictors of poorer mental health during the lockdown [31].

Therefore, the primary aim of the study was to investigate the independent and combined effects of physical activity and sitting time on adults’ mental health with a particular focus on depression, anxiety, and mental wellbeing during the UK lockdown restrictions, including the analysis of potential mediators and confounders of this association.

### Methods

#### Study design

This is a cross-sectional study which utilised an online survey to investigate activity behaviour and mental health status. The study followed guidance from the Strengthening the Reporting of Observational Studies in Epidemiology (STROBE) guidelines [33]. The study received ethical approval from the School of Human & Health Sciences at the University of Huddersfield (application number SREIC/2020/051). Participants were informed at the beginning of the survey that participation was voluntary and provided with further information about the study. Informed consent was implied by completing the questionnaire. Participants who complete the survey were entered into a prize draw of 30 prizes of £50 Amazon vouchers.

#### Participants and setting

We recruited a convenience sample via University staff and student e-mailing list along with University social media advertisements performed by their representatives. In addition, previously identified gatekeepers in Local Authority and Public Health England were asked to support and promote the study through their network, organisation websites, and social media. Inclusion criteria were participants age 18 or over and UK residents.

**Fig. 1:**
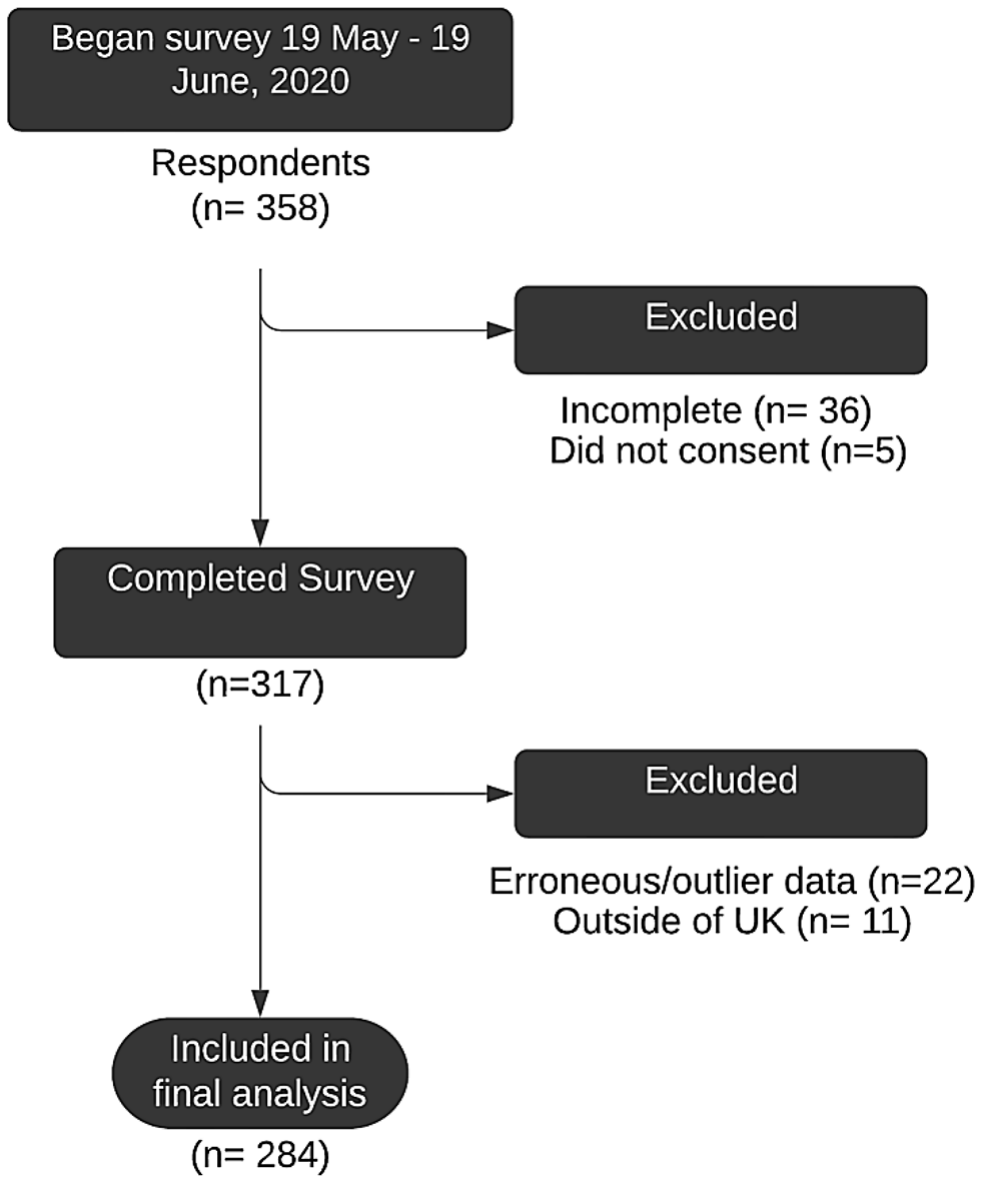
CONSORT diagram of participation

Data were collected during the COVID-19 lockdown in the UK from May to June 2020. During this period, the UK was primarily in Phase 1 of the lockdown in which people were advised to stay at home and just leave the house for shopping for basic necessities, one form of exercise a day, any medical need or to provide care to help a vulnerable person or travelling to and from work if you cannot work from home. However, from 1st of June, the UK moved to Phase 2 in which people from different households were allowed to meet in groups of six, in gardens and outdoor spaces. Figure 1 report the CONSORT diagram of participants.

#### Measurements

An online survey was created using Qualtrics XM survey platform [34] and disseminated online. The survey collected (1) demographic and pre-existing health conditions; (2) habitual physical activity, and (3) mental Health. The demographic data included age, gender, ethnicity, education, socio-economic status, children living in the home, outdoor space, and previous health conditions.

#### Physical activity

Physical activity was measured using the long form of the International Physical Activity Questionnaire (IPAQ) [35], which has been validated [36, 37]. Physical activity output was calculated as metabolic equivalents (MET) per minutes per week in the same activity domains found in the IPAQ (i.e., work-related, transportation, domestic and garden and leisure time). For each type of exercise intensity (walking, moderate, vigorous), the amount of physical activity (days week^–1^) and duration (min day^–1^) was multiplied by an appropriate MET level (1 MET = 3.5 mL kg^–1^ min^–1^) to determine MET·min^–1^ week^–1^ [35]. The data were analysed as a continuous variable MET·min^–1^ week^–1^ and as a categorical variable: (1) Meeting the physical activity guidelines, achieving at least 600 MET min^–1^ week^–1^ and (2) not meeting the physical activity guidelines, less than 600 MET min^–1^ week^–1^. The 600 MET min^–1^ week^–1^ threshold is equivalent to 150 min of moderate-intensity or 75 min of vigorous-intensity aerobic physical activity, throughout the week, which is the international health guidelines recommendation for physical activity [38]. We also used a pragmatic cut-point of 8 h a day to categorise sitting time: (1) low sitting time: < 8 h day^–1^ and (2) high sitting time (≥ 8 h day^–1^). We combined participants according to their physical activity and sedentary behaviour into four groups: (1) high sitting time (≥ 8 h day^–1^) plus low physical activity (< 600 MET min^–1^ week^–1^); (2) high sitting time (≥ 8 h day^–1^) plus moderate or high physical activity (≥ 600 MET ·min^–1^ week^–1^); (3) low or moderate sedentary time (< 8 h day^–1^) plus low physical activity group (< 600 MET ·min^–1^ week^–1^); and (4) low or moderate sedentary time (< 8 h day^–1^) plus moderate or high physical activity (≥ 600 MET min^–1^ week^–1^).

#### Mental health

Mental health was measured by an adapted version of the online UK Biobank mental health questionnaire. This mental health survey that has shown high face validity in relatively “healthy volunteers” [39]. The following domains of the UK Biobank questionnaire were selected to be recorded in this study: (1) screening questions; (2) current depression; (3) current anxiety disorder; and (4) wellbeing.

#### Depression (patient health questionnaire 9-item; PHQ-9)

The PHQ-9 contains nine items and is a well-validated measure of depression [40, 41]. Each item is scored from 0 (not at all) to 3 (almost every day), giving a range of scores from 0 to 27, where a higher score indicates greater depression. Excellent psychometric properties have been established [40, 42]. In the current sample, Cronbach’s alpha was excellent (α = 0.90, Supplement file Table 1).

#### Anxiety (generalized anxiety disorder scale; GAD-2)

For the measure of anxiety, the GAD-2 was used instead of the GAD-7 as it is a brief identification tool designed to minimise completion time but still allow feasibility and accuracy of measurement in this shorter timeframe. The two items are scored from 0 (not at all) to 3 (nearly every day), providing a possible range of scores between 0 and 6. A cut-off of 3 in the GAD-2 scale has been assessed to be the optimal sensitivity to identify GAD, and is advised as the cut-off for the NICE guidelines in anxiety disorder screening [43, 44]. Psychometric properties have been established [45], and in the present sample, Cronbach’s alpha was excellent (α = 0.85, Supplement file Table 2).

#### Subjective wellbeing

The three wellbeing items from the UK Biobank mental health questionnaire were combined to form a total subjective wellbeing score. Each item was scored from 1 (extremely unhappy) to 6 (extremely happy) and included a response option of 0 (don’t know), giving a possible range of scores between 0 and 18, with a higher score indicating greater subjective wellbeing. In the current sample, Cronbach’s alpha was good (α = 0.73, Supplement file Table 3).

#### Previous mental health difficulties

Additionally, two dichotomous variables were created (‘ever diagnosed with mood disorder’ and ‘ever diagnosed with anxiety disorder’) by combining the relevant individual mental health problems asked in the UK Biobank questionnaire.

#### Statistical analysis

Data analysis was performed using IBM SPSS Statistics for Windows (Version 26.0) Descriptive analysis was used to describe the socio-demographic and health data. We used multivariable logistic regression to estimate the combined relationship of sitting time and physical activity adjusted for socio-demographic and health variables. We also explored the correlations between the different physical activity domains (work-related, transportation, domestic and garden and leisure time) with depression, anxiety and wellbeing total score using Spearman correlation coefficient.

Hierarchical linear regression analyses were used to explore the cumulative effects of prominent variables as specified in advance by the review in literature.

For the PHQ-9, GAD-2 and subject wellbeing regression analyses, step 1 consisted of socio-demographic and health variables (age, gender, ethnicity, employment status, income, Covid symptom presence, previous diagnosis of depression or anxiety). Step 2 consisted of weekly MET activity. Step 3 consisted of sitting time. Each step was compared to the previous step, but the coefficients of the model in the final step were interpreted further. Normality was evaluated for each model using a Q-Q scatterplot by comparing the distribution of the residuals with a normal distribution. Normality was assumed as the points formed a relatively straight line. Multicollinearity was evaluated to rule out predictor variables that may be highly correlated with one or more other predictor variables as this can affect the interpretation of the regression coefficients [46]. Variance Inflation Factors (VIF) greater than 5 are cause for concern [47], however, for all three steps in the model, all predictors have VIFs less than 10. To identify influential outliers, Studentized residuals were calculated, and the absolute values were plotted against the observation numbers. Multivariate outliers were removed before analysis using Mahalanobis distances, with the critical chi-square value at alpha 0.001 selected [48]. These were assumed to be errors outliers [49], since there was no data entry or coding errors (i.e., data were directly exported from Qualtrics XM survey to SPSS).

### Results

A total of 284 participants were included in the analysis. Incomplete, invalid, and ineligible responses were not retained for analysis, representing a completion rate of 88.5% with 79.3% being analysed after exclusions.

The average age of participants was 33.5 ± 12.4 years. Participants’ characteristics are described in Table 1. Briefly, most of the sample constituted of females, from white ethnicity, higher income (over £40 k year^−1^), and with no pre-existing health conditions.

**Table 1:** Participants demographics consisting of gender, ethnicity, employment status, level of education, children in the house, outdoor space, and previous health condition

Variable	*n*	*%*
Sex		
Female	208	74.8
Male	68	24.5
Ethnicity		
White	222	79.9
Minority ethnicity	55	19.8
Employment status		
Student or economically inactive	129	46.4
Employed	147	52.9
House income		
Over £40 k per year	108	38.8
£30-£40 k per year	47	16.9
£20-£30 k per year	36	12.9
£10-£20 k per year	35	12.6
Below £10 k per year	48	17.3
Children in house		
No children	182	65.5
Children	96	34.5
Outdoor space		
Yes	244	87.8
No	34	12.2
Presence of health condition		
One or more health condition	99	35.6
No health condition	174	62.6
COVID symptom presence		
Yes	46	16.2
No	238	83.8
Previous diagnosis of mood or anxiety disorder		
Mood disorder	75	26.4
Anxiety	77	27.1

COVID-19 symptoms considered here are high temperature, a new continuous cough and loss or change to sense of smell or taste

We have also explored the effect of stage of lockdown on participants’ outcome. Most participants, 78%, took part in the study when the UK was in Phase I of the lockdown (up to 31^st^ of May) while 22% of the responses were collected from 1 June (Phase II). A series of t-tests revealed no significant differences in PA, siting time, depression, anxiety or wellbeing scores between the two periods of lockdown. Lockdown period was therefore not further imputed in any analysis.

Table 2 presents the combined analysis of physical activity and sitting time. Participants who reported low sitting time and low physical activity and those who reported low sitting time with moderate or high physical activity showed significantly lower depression score compared to participants with high sitting time and low physical activity (reference group). Similarly, participants with low sitting time and moderate or high physical activity had a significantly higher wellbeing score compared to those with high sitting time and low physical activity.

**Table 2:** Combined effects of sedentary behaviour and physical activity on anxiety, depression, and wellbeing

	*N*%	Depression			Anxiety			Wellbeing		
		*B*	CI	*p*	*B*	CI	*p*	*B*	CI	*p*
High sitting time and low physical activity.	65 (25)	Reference			Reference			Reference		
High sitting time and moderate or high physical activity.	53 (20)	− 0.41	− 2.591.7	0.713	0.46	− 0.56, 1.48	0.374	0.26	− 0.72, 1.24	0.608
Low sitting time and low physical activity.	51 (19)	− 2.7	− 4.88, − 0.52	0.016*	− 0.09	− 1.11, − 0.93	0.863	0.65	− 0.33, 1.63	0.196
Low sitting time and moderate or high physical activity.	95 (36)	− 2.9	4.74, − 1.06	− 0.001*	− 0.07	− 0.92, 0.79	0.88	1.23	0.41, 2.06	0.004

High sitting time ≥ *8 h day*; low sitting time < 8 h day; moderate or high physical activity ≥ 600 MET·min^–1^ week^–1^; low physical activity < 600 MET·min^−1^ week^−1^

Table 3 reports the association between the different domains in physical activity (i.e., work domain, transportation domain, domestic and garden domain and leisure-time) with depression, anxiety and wellbeing total score. There was a negative and significant association between depression score and domestic and garden physical activity and leisure-time physical activity domains. However, there was a significant and positive association between these same domains and wellbeing score. A similar significant positive association was noted between the work-related physical activity domain and wellbeing score.

**Table 3:** Association between work-related, on depression, anxiety, and wellbeing scores

	*n* = 269	Total depression Score	Total anxiety score	Total subjective Wellbeing score	
Work related PA	*r*_s_ (95% CI)	− 0.009 (− 0.21, 0.03	− 0.04 (− 0.16, 0.08)	0.016 (0.05, 0.28)	
	*p* value	0.15	0.553	0.007*	
Transportation PA	*r*_s_ (95% CI)	− 0.07 (− 0.19, − 0.05)	− 0.02 (− 0.1, 0.13)	0.08 (− 0.04, 0.2)	
	*p* value	0.257	0.799	0.171	
Domestic and garden PA	*r*_s_ (95% CI)	− 0.13 (− 0.25, − 0.01)	− 0.08 (− 0.2, − 0.04)	0.014 (0.02, 0.25)	
	*p* value	0.031*	0.165	0.024*	
Leisure-time PA	*r*_s_ (95% CI)	− 0.16 (− 0.27, − 0.04)	− 0.06 (− 0.18, − 0.06)	0.17 (0.05, 0.28)	
	*p* value	0.009*	0.309	0.006*	

*Statistically significant

Results from the hierarchal regression analysis was performed in three steps for each mental health outcome are detailed below.

#### Depression

The socio-demographic and health variables included in Step 1 analysis explained 33% of the variance of the depression score, *F*(11, 221) = 9.70, *p* < 0.001. With the addition of weekly activity measured in Step 2 there was a statistically significant increase in variation of 1.5% (*F* change (1, 220) = 5.05, *p* = 0.026). Finally, in Step 3, there was a further statistically significant increase in 8% of the variance with the inclusion of sitting time (*F* change (1, 219) = 30.89, *p* < 0.001). However, weekly activity variable becomes non-significant when the sitting time was added in the model. The final model was statistically significant, *F* (13, 219) = 12.31, *p* < 0.001, and explained 42% of the variance in the PHQ-9 depression scores. Younger age, female, on lower income, with one or more comorbid health conditions, previous diagnosis of mood disorder and increased sitting time independently correlated with higher depression scores (Table 4).

**Table 4:** Hierarchical multiple regression analysis for correlates of depression scores

	*R*^2^	*R*^2^ change	β	*B*	SE	CI 95% (*B*)
Step 1	0.33***					
Age			− 0.22**	− 0.12	0.04	− 0.19/− 0.04
Gender			− 0.11	− 1.63	0.85	− 3.31/0.06
Ethnicity			0.13*	2.17	0.97	0.26/4.09
Employment			0.00	0.01	0.94	− 1.84/1.86
Income > 40 k						
< 10 k			0.18*	3.15	1.31	0.58/5.73
10–20 k			0.08	1.49	1.30	− 1.06/4.04
20–30 k			0.08	1.55	1.21	− 0.84/3.94
30–40 k			0.06	0.98	1.03	− 1.05/3.00
Comorbid health condition			0.24***	3.10	0.83	1.47/4.72
COVID symptoms			0.08	1.38	0.97	− 0.53/3.30
Ever diagnosed mood disorder			0.26***	3.59	0.88	1.86/5.33
Step 2	0.34***	0.01				
Age			− 0.21**	− 0.11	0.04	− 0.18/− 0.04
Gender			− 0.10	− 1.52	0.85	− 3.19/0.15
Ethnicity			0.11	1.75	0.98	− 0.18/3.69
Employment			0.02	0.23	0.94	− 1.61/2.08
Income > 40 k						
< 10 k			0.18*	3.09	1.29	0.54/5.64
10–20 k			0.10	1.84	1.29	− 0.71/4.40
20–30 k			0.08	1.50	1.20	− 0.71/3.87
30–40 k			0.06	0.93	1.02	− 1.08/2.93
Comorbid health condition			0.23***	2.96	0.82	1.34/4.57
COVID symptoms			0.20	1.76	0.98	− 0.16/3.69
Ever diagnosed mood disorder			0.25**	3.54	0.87	1.82/5.26
Physical activity			− 0.13*	− 0.04	0.02	− 0.08/− 0.01
Step 3	0.42***	0.08				
Age			− 0.18**	− 0.09	0.03	− 0.16/− 0.03
Gender			− 0.13*	− 1.91	0.80	− 3.48/− 0.33
Ethnicity			− 0.07	1.10	0.93	− 0.73/2.93
Employment			0.04	0.51	0.88	− 1.23/2.24
Income > 40 k						
< 10 k			0.16*	2.87	1.21	0.48/5.26
10–20 k			0.08	1.57	1.22	− 0.83/3.96
20–30 k			0.08	1.50	1.13	− 0.72/3.72
30–40 k			0.04	.66	0.96	− 1.22/2.55
Comorbid health condition			0.22***	2.82	0.77	1.30/4.33
COVID symptoms			0.06	1.00	0.93	− 0.82/2.83
Ever diagnosed mood disorder			0.22**	3.05	0.82	1.43/4.67
Physical activity			0.01	0.00	0.02	− 0.04/0.04
Sitting time			0.33***	0.01	0.00	0.01/0.01

Statistical significance: *p < 0.05, **p < 0.01, ***p < 0.001

#### Anxiety

Step 1 of the analysis explained 31% of variance in the GAD-2 scores, *F*(11, 221) = 8.95, *p* < 0.001. The inclusion of weekly activity did not make a statistically significant contribution to the model (*F* change (1, 220) = 0.00, *p* = 0.99). The inclusion of sitting time at Step 3 explained an additional statistically non-significant 1% of the variance in anxiety, *F* change (1, 219) = 3.31, *p* = 0.08. The final model was statically significant, *F*(13, 219) = 7.84, *p* < 0.001, and explained 32% of variance in the GAD-2 scores. Being younger, female, on lower income, having one or more comorbid health condition, and the previous diagnosis of anxiety disorder were statistically significant independent correlates of anxiety (Table 5).

**Table 5:** Hierarchical multiple regression analysis results for correlates of anxiety scores

	*R*^2^	*R*^2^ change	β	*B*	SE	CI 95% (*B*)
Step 1	0.31***					
Age			− 0.19**	− 0.03	0.01	− 0.05/− 0.01
Gender			− 0.24***	− 1.07	0.26	− 1.59/− 0.56
Ethnicity			− 0.02	− 0.12	0.29	− 0.70/0.46
Employment			0.03	0.12	0.29	− 0.45/0.68
Income > 40 k						
< 10 k			0.15*	0.80	0.39	0.03/1.58
10–20 k			0.10	0.59	0.39	− 0.18/1.36
20–30 k			0.05	0.30	0.37	− 0.43/1.02
30–40 k			0.07	0.37	0.31	− 0.25/0.98
Comorbid health condition			0.16**	0.63	0.23	0.17/1.09
COVID symptoms			− 0.01	− 0.03	0.30	− 0.61/0.55
Ever diagnosed anxiety disorder			0.26***	1.10	0.25	0.61/1.59
Step 2	0.31***	0.00				
Age			− 0.19**	− 0.03	0.01	− 0.05/0.01
Gender			− 0.24***	− 1.07	0.26	− 1.59/− 0.56
Ethnicity			− 0.02	− 0.12	0.30	− 0.71/0.48
Employment			0.03	.12	0.29	− 0.45/0.68
Income > 40 k						
< 10 k			0.15*	0.80	0.39	0.03/1.58
10–20 k			0.10	0.59	0.40	− 0.19 /1.37
20–30 k			0.05	0.30	0.37	− 0.43/1.02
30–40 k			0.07	0.37	0.31	− 0.24 /− 0.25
Comorbid health condition			0.16***	0.63	0.24	0.16 /1.09
COVID symptoms			− 0.01	− 0.03	0.30	− 0.62/0.56
Ever diagnosed anxiety disorder			0.26***	1.10	0.25	0.60/1.59
Physical activity			0.00	− 02.56	0.01	− 0.01/.01
Step 3	0.32***	0.01				
Age			− 0.18*	− 0.03	0.01	− 0.05/− 0.01
Gender			− 025***	− 1.12	0.26	− 1.63/− 0.60
Ethnicity			− .04	− 0.18	0.30	− 0.77/0.41
Employment			0.04	0.14	0.29	− 0.43/0.71
Income > 40 k						
< 10 k			0.15*	0.77	0.39	− 0.00/1.55
10–20 k			0.10	0.77	0.39	− 0.22/1.34
20–30 k			0.05	0.30	0.37	− 0.43/1.02
30–40 k			0.07	0.34	0.31	− 0.28/.95
Comorbid health condition			0.15*	0.60	0.23	0.14/1.06
COVID symptoms			− .02	-.10	.30	− 0.70/0.49
Ever diagnosed anxiety disorder			0.25**	1.05	.25	0.56/1.55
Physical activity			0.05	.01	.01	0.02/− 0.03
Sitting time			0.11	.00	.00	0.00/0.14

Statistical significance: *p < 0.05, **p < 0.01, ***p < 0.001

#### Subjective wellbeing

The sociodemographic and health variables explained 22% of the variance in the subjective wellbeing scores, *F*(12, 220) = 5.03, *p* < 0.001. The inclusion of weekly activity at Step 2 explained an additional statistically significant 1% variance in wellbeing scores, *F* change (1, 219) = 4.60, *p* = 0.03. The inclusion of sitting time at Step 3 added an additional 4% of the variance and was statistically significant, *F* change (1, 218) = 11.67, *p* = 0.001. The inclusion of sitting time rendered physical activity non-significant. The final model was statistically significant, *F* (14, 218) = 5.77, *p* < 0.001 and explained 27% of the variance in the subjective wellbeing scores. Higher wellbeing scores were independently associated with being healthy (i.e., no comorbid illness), no previous diagnosis of a mood disorder and less time sitting (Table 6).

**Table 6:** Hierarchical multiple regression analysis for correlates of wellbeing scores

	*R*^2^	*R*^2^ change	β	*B*	SE	CI 95% (*B*)
Step 1	0.22***					
Age			0.12	0.03	0.02	− 0.01/0.06
Gender			− 0.01	− 0.08	0.37	− 0.82/0.65
Ethnicity			0.02	0.13	0.42	− 0.71/0.96
Employment			− 0.02	− 0.08	0.41	− .89/0.73
Income > 40 k						
< 10 k			− 0.16	-− 1.10	.57	− 2.22/0.01
10–20 k			− 0.01	− 0.04	0.56	1.15/1.07
20–30 k			− 0.02	− 0.12	0.53	− 1.15/0.92
30–40 k			− 0.06	− 0.40	0.45	− 1.28/0.48
Comorbid health condition			− 0.25***	− 1.30	0.36	− 2.01/0.59
COVID symptoms			0.01	0.05	0.42	− 0.78/0.89
Ever diagnosed mood disorder			− 0.26***	− 1.47	0.48	− 2.27/− 0.66
Ever diagnosed anxiety disorder			.07	0.37	0.38	− 0.39/1.12
Step 2	0.23***	0.01				
Age			0.11	0.02	0.02	– 0.01/.05
Gender			–0.02	– 0.13	0.37	– 0.87/.60
Ethnicity			0.05	0	0.43	– 0.54/1.14
Employment			–0.04	– 0.18	0.41	– 0.99/.63
Income > 40 k						
< 10 k			– 0.15	– 1.08	0.56	– 2.19/.03
10–20 k			– 0.02	– .19	0.56	– 1.30/.93
20–30 k			– 0.01	– .09	0.52	– 1.13/.94
30–40 k			– 0.06	– .38	0.44	– 1.25/.49
Comorbid health condition			− 0.24***	− 1.24	0.36	− 1.62/− 0.53
COVID symptoms			− 0.01	− 0.10	0.43	− 0.94/0.74
Ever diagnosed mood disorder			− 0.25***	− 1.43	0.41	− 2.23/0.63
Ever diagnosed anxiety disorder			0.06	0.34	0.38	− 0.41/1.09
Physical activity			0.13*	0.02	0.01	0.00/0.04
Step 3	0.27***	0.04				
Age			0.09	0.02	0.02	− 0.01/.05
Gender			− 0.00	− 0.02	0.37	− 0.73/.70
Ethnicity			0.07	0.49	0.42	− 0.34/1.31
Employment			− 0.05	− 0.24	0.40	− 1.03/0.55
Income > 40 k						
< 10 k			− 0.14	− 1.01	0.55	− 2.10/0.07
10–20 k			− 0.02	− 0.11	0.55	− 1.20/0.97
20–30 k			− 0.01	− 0.10	0.51	− 1.10/0.91
30–40 k			− 0.05	− 0.30	0.43	− 1.16/0.55
Comorbid health condition			− 2.30***	− 1.21	0.35	− 1.90/− 0.52
COVID symptoms			0.02	0.10	0.42	− 0.73/0.93
Ever diagnosed mood disorder			− 0.24***	− 1.33	0.40	− 2.11/− 0.55
Ever diagnosed anxiety disorder			0.08	0.43	0.37	− 0.30/1.17
Physical activity			0.04	0.01	0.01	− 0.01/0.02
Sitting time			− 0.23***	− 0.00	0.00	− 0.00/− 0.00

Statistical significance: *p < 0.05, **p < 0.01, ***p < 0.001

### Discussion

The primary aim of this study was to explore the effect of physical activity and sitting time on participants’ mental health during the UK COVID-19 lockdown restrictions. The study found that key socio-demographic, health outcomes and sitting time explained 42% of the depression score variation, and in particular that physical activity did not account for the variation in depression score when sitting time was added in the model. The same findings were confirmed by the analysis of the combined association, which showed that those with lower sitting time had a significantly lower depression score in participants with either low or moderate/high physical activity level. Similar results were found for wellbeing where socio-demographic, health outcomes and sitting time influenced the subjective wellbeing (27% variance) with physical activity becoming non-significant when sitting time were taken into account. However, the combined analysis revealed that wellbeing was significantly higher in the group with low sitting time and moderate or high physical activity combined compared to the reference group (i.e., high sitting time and low physical activity). Finally, only socio-demographic and health outcomes contributed to the variation in anxiety score (32% variance).

The lockdown restriction has caused serious societal impacts that affected population behaviour and psychological aspects. Loneliness has increased significantly in the population, and it appeared to be maintained despite the reopening of communities [50]. Our study found that sitting time had a significant impact on depression and wellbeing. Others have highlighted the importance of reducing sedentary behaviour for mental wellbeing during COVID-19 isolation [51], which is reinforced by the literature as there is strong evidence from a meta-analysis of observational studies that sedentary behaviour is positively associated with the risk of depression [52]. However, the association between wellbeing and sedentary behaviour is controversial [53, 54], and sedentary behaviour appears to have only a small effect on anxiety [55, 56].

Most studies have focused on COVID-19 restrictions and the effect of physical activity on mental health [27, 27, 31], while some have highlighted the importance of sedentary behaviour [29, 58, 59]. Studies have noticed an increase in sedentary behaviour compared to pre-pandemic [29, 60], with some studies showing that prolonged sitting (more than 10 h day^−1^) was associated with depressive symptoms [61]. This has similarity to our research which found that participants who sat for 8 or more hours a day had higher depression and anxiety scores and lower wellbeing, compared to participants who sat for a shorter period. In another study, sitting and screen time were also compared among participants who were considered active and inactive pre-COVID-19 restrictions [29]. The research found that an increase in screen time and reduction in physical activity was associated with higher depressive symptoms and lower positive mental health in general [29]. The same findings were observed in a Brazilian population where a decrease in unhealthy movement behaviour (i.e., inactivity and sedentary behaviour) was associated with loneliness, sadness and anxiety during COVID-19 pandemic [62].

Although the association between sedentary behaviour and mental health during lockdown have been studied, from our knowledge, no studies have investigated the moderation effect of physical activity on the impact of sedentary behaviour on mental health outcomes. There is evidence that higher volumes of physical activity (60–75 min day^−1^) are protective of the increased risk of mortality from prolonged sitting (> 8 h day^−1^) [63–65]. However, the mediating effect from other health outcomes and in particular mental health is less clear [66, 67]. Using compositional analysis of associations [68], a study found that self-reported mental health measured by a single mental health question, was significantly associated with physical activity in older adults (65–79 years). However, sedentary behaviour did not impact on mental health in young (18–64 years) or older age group (65–79 years) [69]. This is in contradiction to the findings of our study, with a younger population (33.5 ± 12.4 years), which used a more comprehensive assessment of mental health (UK Biobank mental health assessment). However, physical activity and sitting time were self-reported in our study, rather than objectively measured compared with previous study [68]. The type of measurement might have affected the association outcome. The analysis of data from the Health Survey for England revealed that only objectively measured light-intensity activity was associated with a lower risk of psychological distress rather than self-reported data. However, sedentary time was associated with adverse mental health, independently if measured objectively or self-reported [70].

This study found only a minimum effect of physical activity on depression score, which turned out to be non-significant when sitting time was imputed into the model. This was further supported by the combined analysis of the effect of sitting time and physical activity (Table 2). The sub analysis which examined the relationship between different domains of physical activity and mental health outcomes revealed that domestic and garden physical activity and leisure-time physical activity domains were negatively associated with depression and positively associated with wellbeing. Similarly, the work-related domain was positively correlated with wellbeing. However, these results need to be interpreted with caution as the overall effect of physical measured by the mixed model was not significant and small sample size, increasing the possibility of a Type I error.

A study with a large cohort of Australian women, also found that domestic and garden work were positively correlated with physical and mental wellbeing in mid-aged (50–55 years) and older women (76–81 years) but negatively associated with younger women (25–30 years) [71].Our study consisted primarily of women (74.8%) at a younger age (33.5 ± 12.4 years). However, our study was conducted during lockdown restrictions in which the opportunities to do physical activity were very restricted. Gardening, in particular, showed a positive impact on psychopathological distress, in a study conducted in Italy, during the Covid-19 lockdown [72]. Concerning leisure-time there is strong evidence from a meta-analysis that leisure-time physical activity positively affects mental health [73]. However, the same study showed ambiguous results concerning work-related physical activity as a significant positive association was observed with mental health but also with mental ill-health, which the authors justify as variation in the population between the different studies. In our study, the opportunity to go out to work and do a labour job which requires moderate or vigorous levels of physical activity, during lockdown, might have had positive impact of participants wellbeing giving a sensation of freedom and normality, but again caution in interpretation of these findings are needed.

Finally, all model analysis has shown an association between particular socio-demographic outcomes and pre-existing conditions on mental health domains. Common socio-demographic predictors for depression and anxiety were young and female. The main course of recruitment in this study were university students. It is known that university students have higher rates of depression compared to the general population [74]. It is also known that women are twice more likely than men to suffer from depression, with the increased risk persisting until the mid-50 s [75]. Likewise, anxiety disorders are more prevalent and disabling in women compared to men [76]. Other factors that were associated with mental health problems in this study were lower-income, with one or more comorbid health conditions or previous diagnosis of mental health disorder. This is echoed by other studies which explored the predisposing factors of mental health problems during COVID-19 pandemic [77].

This study has a few limitations, including the cross-sectional design which precludes us from making inferences on causality. Likewise, the use of self-reported physical activity has low validity compared to objective measures of physical activity and sedentary (i.e., accelerometers) [78]. However, the use of objective measures is not viable, especially in lockdown circumstances. Some considerations need to be taken when interpreting the findings of this study. The sample consisted primarily of healthy and active individuals as 78% of our sample meet the recommendations for physical activity. However, 55% of our sample reported a sitting time of more than 8 h a day. This sufficiently active but sedentary at the same time might have skewed our findings compared to studies which recruited a primarily inactive population during COVID-19 lockdown restrictions. Likewise, although we performed further analysis to explore the association of different physical activity domains with mental health outcomes, the same analysis could not be performed for sedentary behaviour since the IPAQ questionnaire does not contain questions on domains of sedentary behaviour. There is evidence suggesting that type sedentary behaviour might have a different impact on mental health, with TV viewing, for example, having a negative effect while reading and working showing no association or a positive effect [79, 80]. Future studies should investigate the impact of different sedentary behaviour activities on mental health outcomes. Likewise, our sample constituted primarily of females (74.8%), limiting our ability to generalise the findings to a mixed-gender population. The reason for more females in our sample is perhaps because women are more likely than men to respond to research surveys [81]. Also, our recruitment focused primarily on University staff and students from the School of Health, who are predominately female. However, the study has some strengths, including the use of a comprehensive assessment of mental health and a robust statistical analysis with adjustment of socio-demographic and health cofounders.

### Conclusion

During strict lockdown in the UK, the government underlined the importance of being physically active by allowing people to exercise as one of the only reasons they could leave their homes. This policy recommendation is welcome, considering the plethora of benefits of physical activity on health and wellbeing. However, the findings from this study and others from the literature, have revealed that reducing sedentary behaviour or sitting time might have a positive effect on individuals mental health, suggesting that public health recommendations should encourage the reduction of sitting time for mental health benefits. The advocate of reducing sitting time might be easier to implement in individuals or populations with mobility impairment or functional disability.
